# Supplementary material for: Amphibious epidermal area networks for uninterrupted wireless data and power transfer
Source: Nat Commun. 2023 Nov 18;14:7522. doi: 10.1038/s41467-023-43344-6 (PMC10657464; doi:10.1038/s41467-023-43344-6)
Supplement: Supplementary file 1 — Supplementary Information [file 41467_2023_43344_MOESM1_ESM.pdf]

Supplemental Material:

Amphibious Epidermal Area Networks for Uninterrupted Wireless Data and Power Transfer

Amirhossein Hajiaghajani, et al.

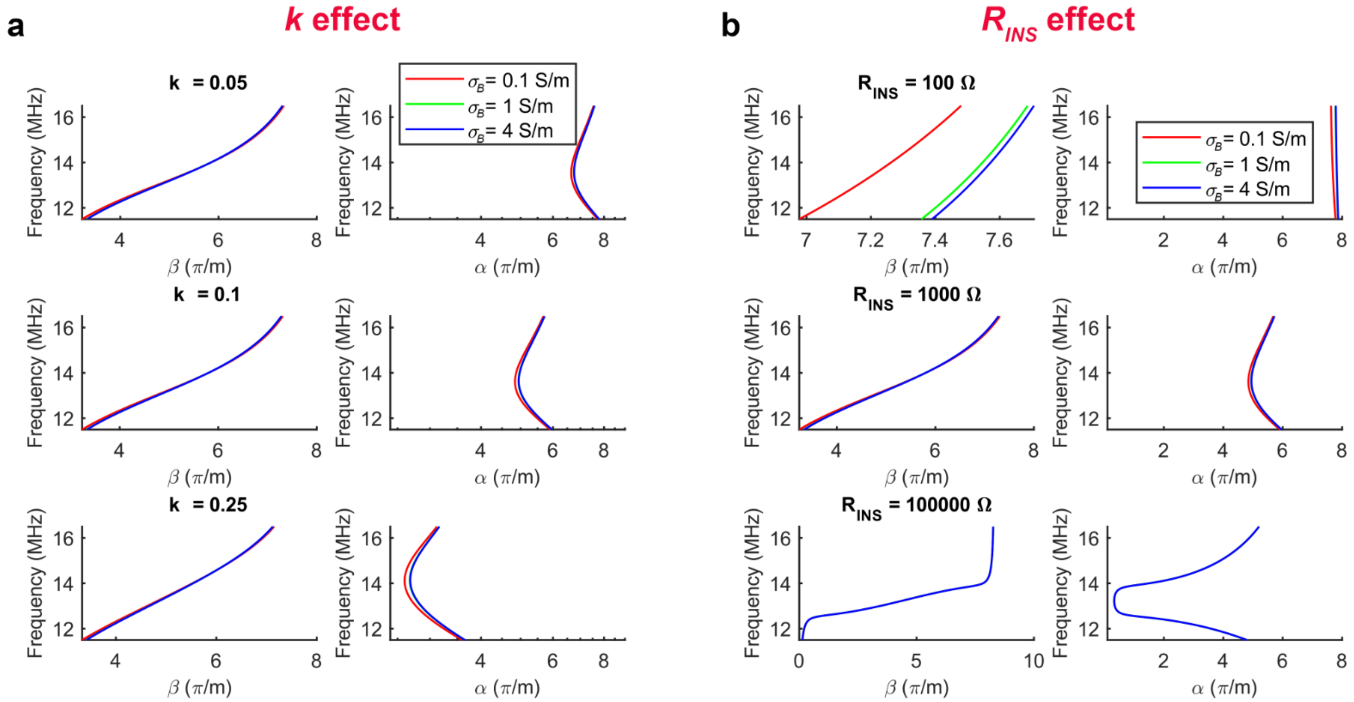

Supplementary Fig. 1. Comparison of dispersion diagrams for variable background media conductivity (σ_B), mutual coupling factor (k), and insulator's effective resistance (R_{INS}) which depends on the area exposed to lossy medium in addition to the insulator's conductivity.

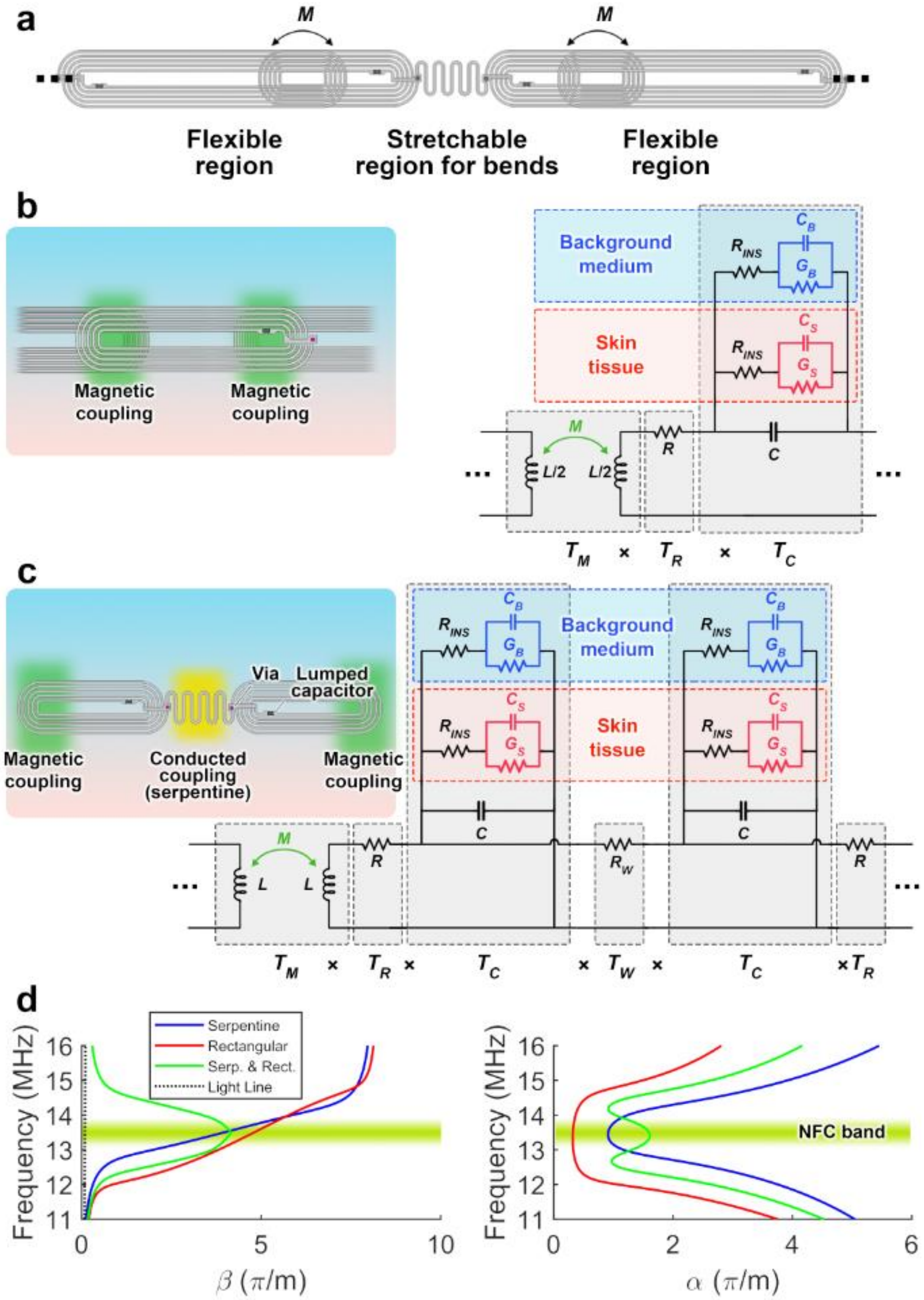

Supplementary Fig. 2. Conventional and complex resonator analysis. (a) An MI array here may consist of (b) magnetically- or (c) conductively-coupled resonators. (d) The dispersion properties of the conductively coupled resonators are modeled by breaking the unit cell resonators into cascaded ABCD transfer matrices and calculating the eigenvalues to obtain the dispersion diagrams. The insulator is assumed to possess sufficient electrical resistance (R_{INS}>10 kΩ) to block the stray electric fields from leaking currents through the skin and lossy background media. This can be practically achieved by utilizing a thing encapsulation.

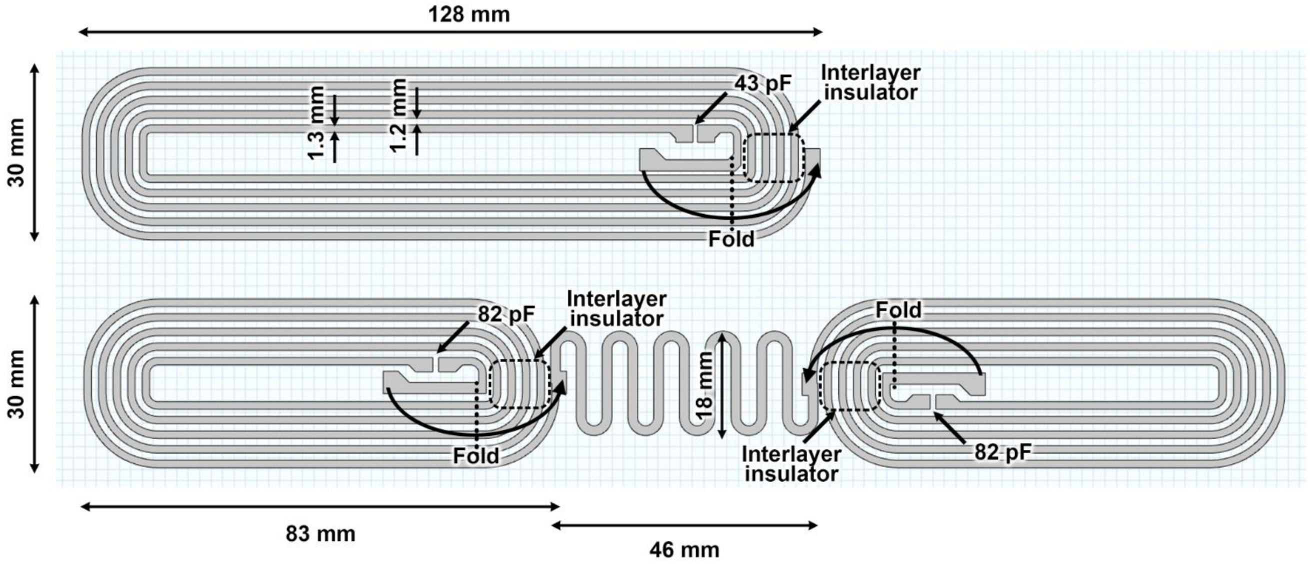

Supplementary Fig. 3. The coil geometries and tuning capacitors for magnetic- and conducted-coupling structures resonating at NFC carrier frequency of 13.56 MHz.

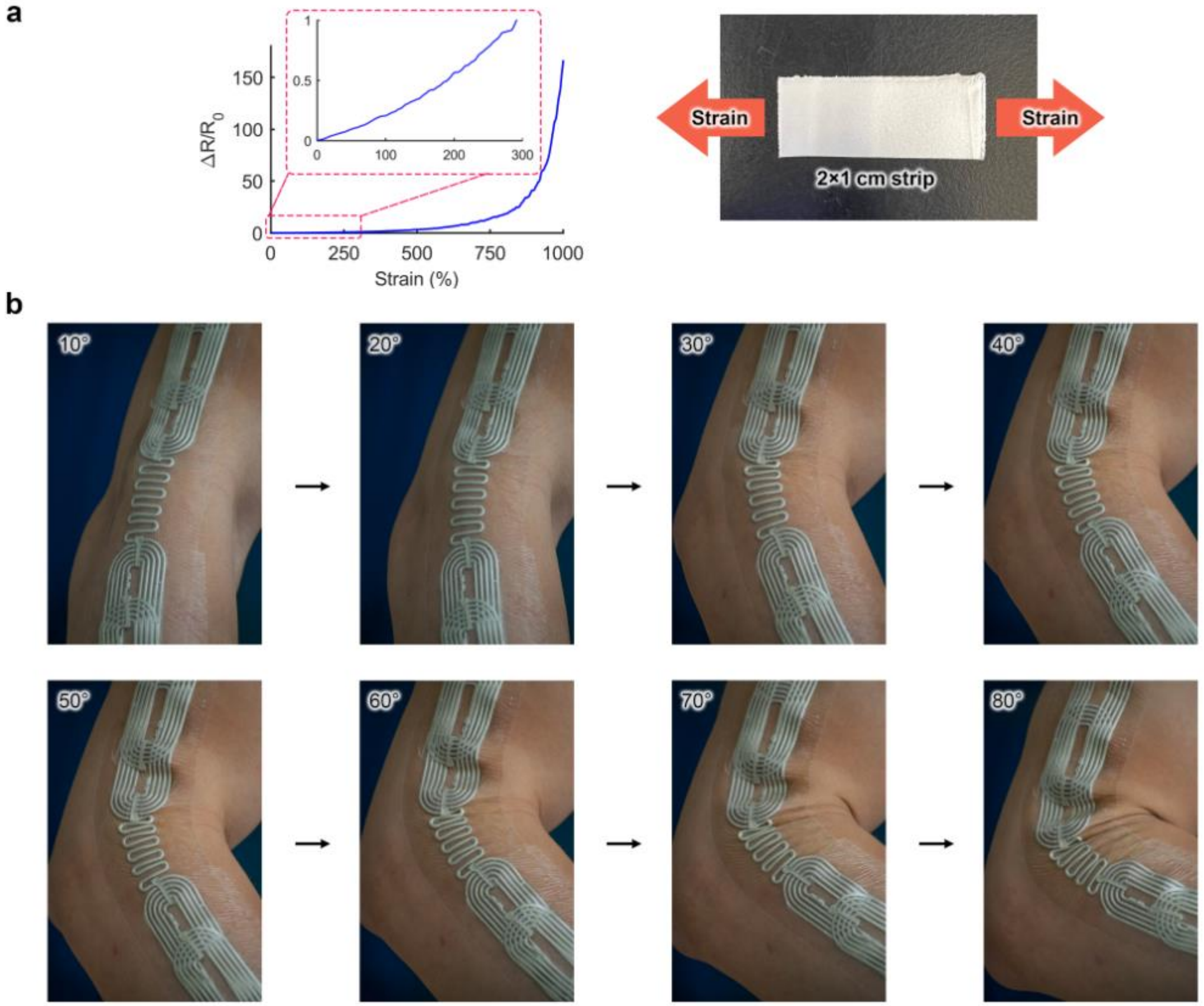

Supplementary Fig. 4. Stretchability of the (a) strip-shaped and (b) resonator geometry during bending phases. Utilizing the trace-coupled resonator (through the serpentine structure) enables absorbing the bending strain and applies minor strain to the coils.

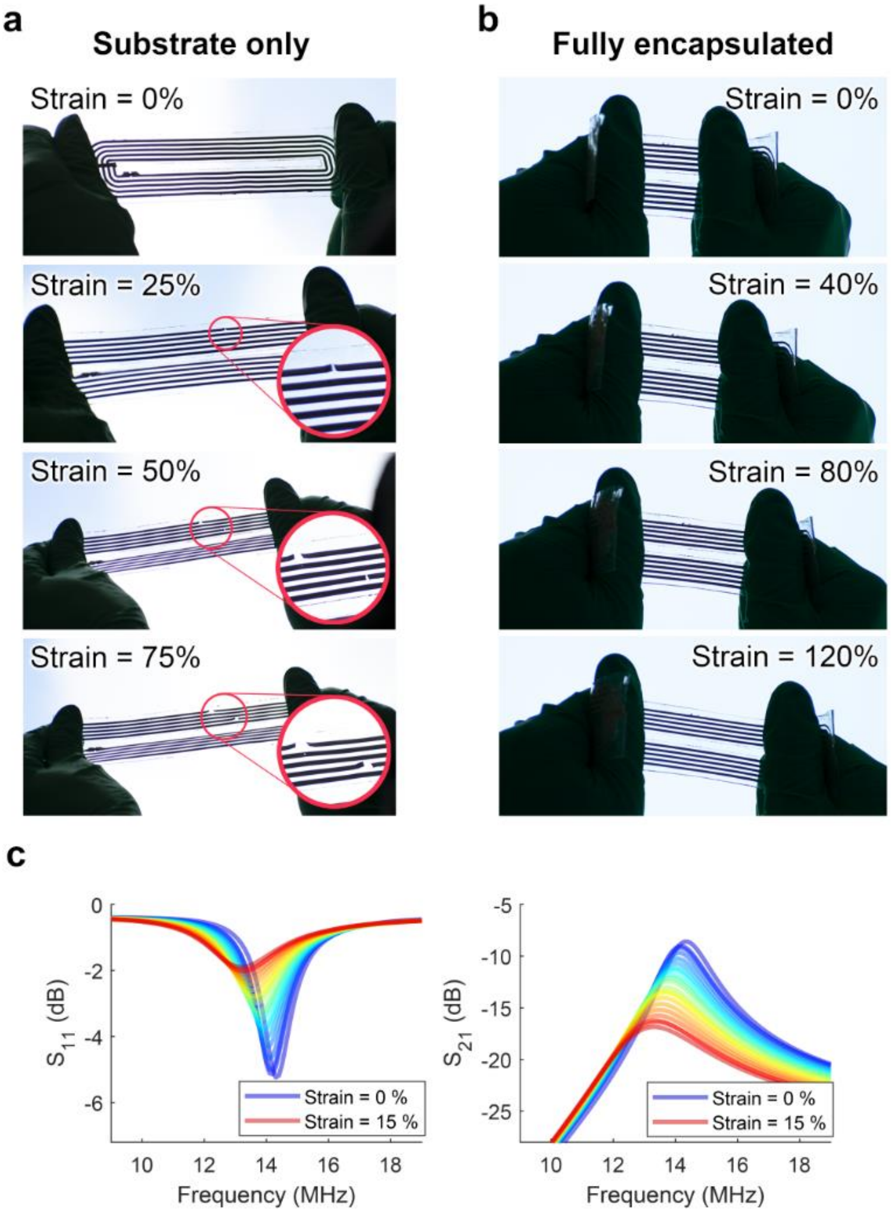

Supplementary Fig. 5. Assessing the stretching durability of the ink under two conditions: (a) without PDMS encapsulation, the tuning capacitor junction did not fail for up to 100% strain. The top insulator is removed to show the failure points; (b) with complete PDMS encapsulation. In both cases, a 0.5 mm thick PDMS substrate was employed, along with PDMS top sealing in the fully encapsulated scenario. The comprehensive PDMS encapsulation significantly enhances the stretching threshold, increasing it from below 25% to above 120%. (c) The measured transmission loss for substrate-only configuration represents minor frequency shift in addition to added Ohmic loss due to temporary low concentration of silver flake under stretching.

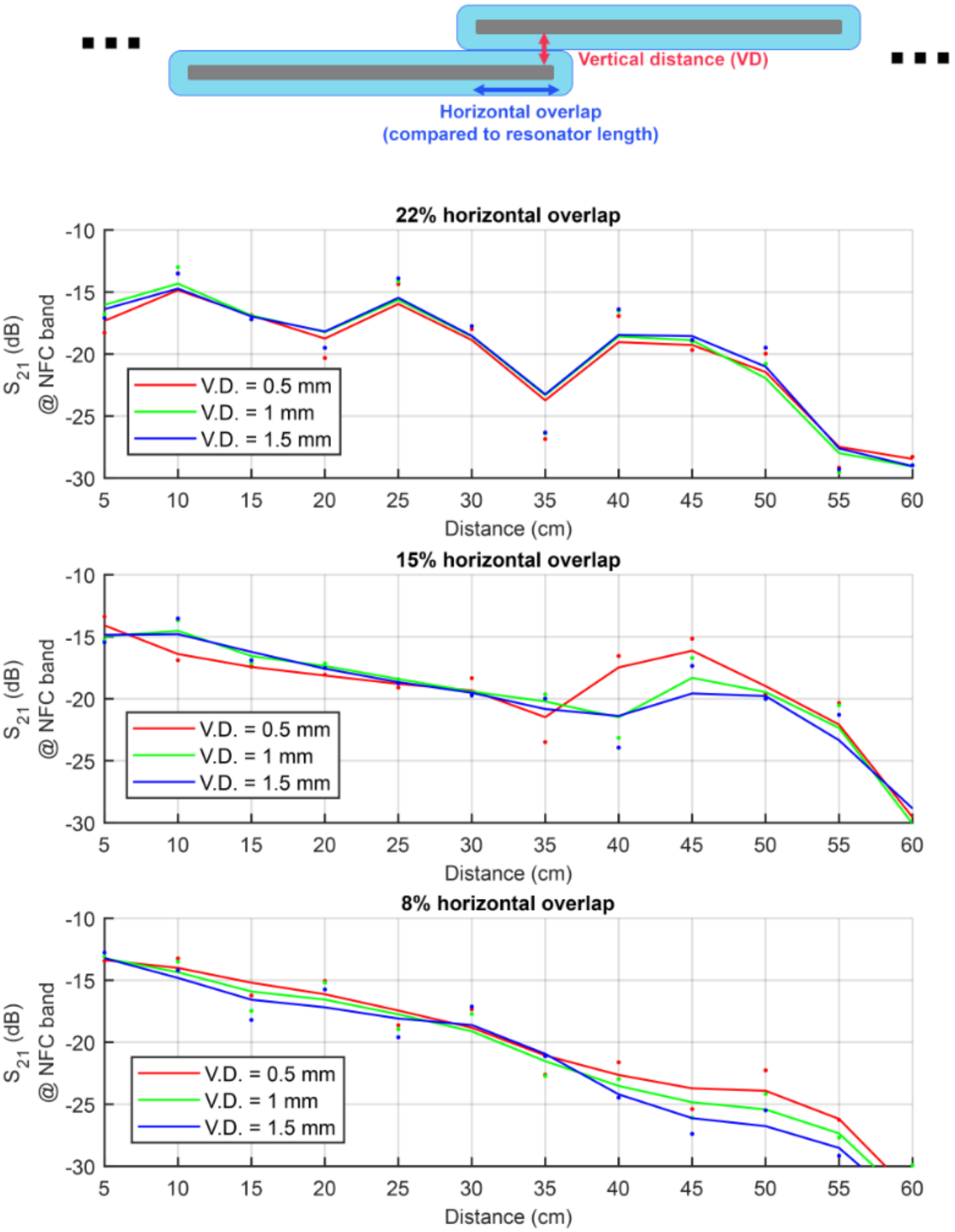

Supplementary Fig. 6. Effect of vertical distance (VD, resulted by encapsulating PDMS thickness) on transmission profile of the MI array. For large enough horizontal overlaps (to induce any transmission passbands), the vertical distance does not impact the transmission profile significantly. Greater coupling factors (i.e. larger horizontal overlap and smaller vertical distance) contributes to larger standing wave ratios that is eventually observed in form of transmission local nulls at certain areas. These artifacts, however, are yet above the threshold for an end-to-end successful NFC communication.

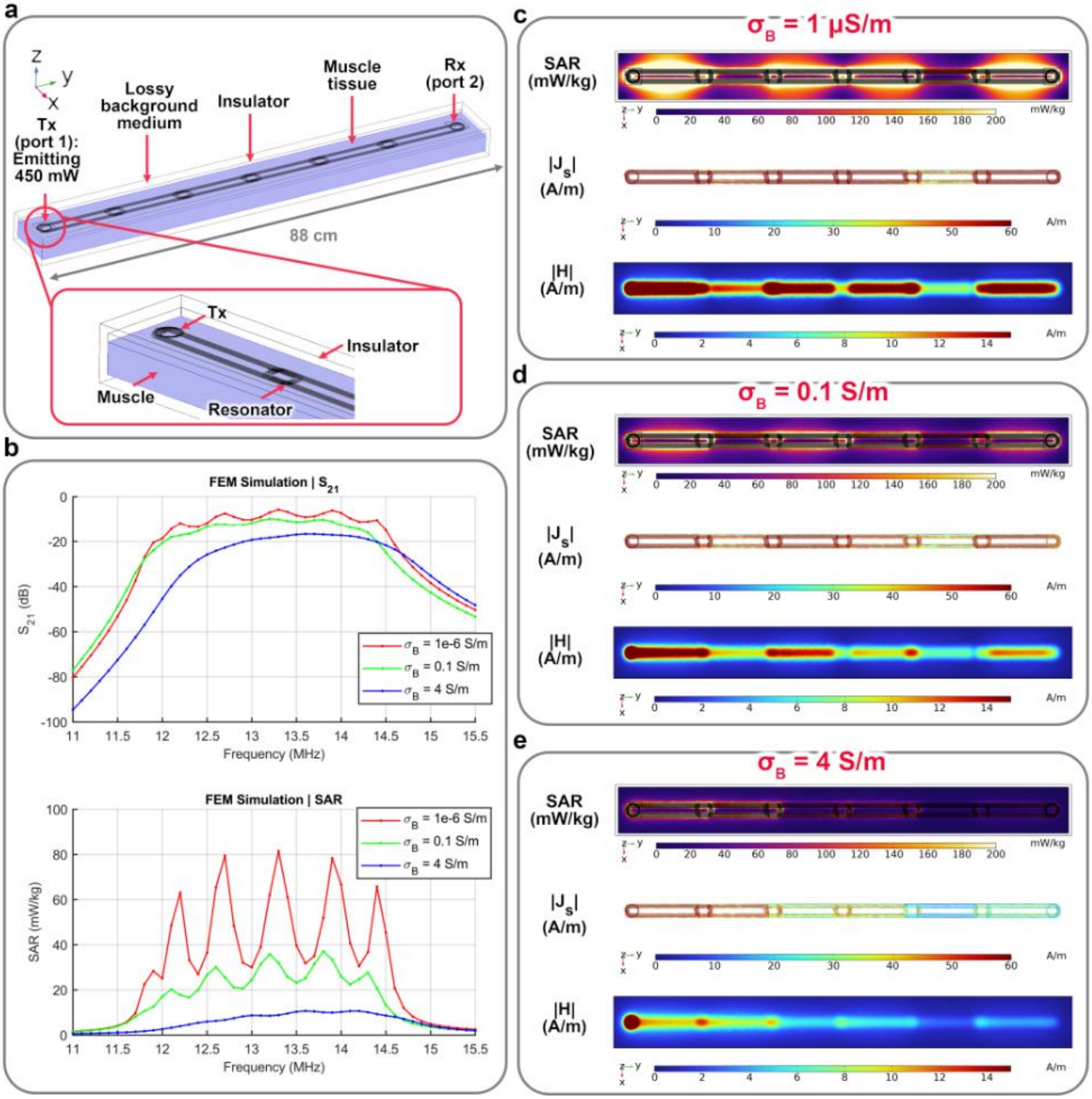

Supplementary Fig. 7. Finite element method simulations demonstrating SAR and transmission in various lossy background medium settings. (a) The simulation setup includes insulated metamaterials on top of the muscle tissue (density of 1090 kg/m^3, permittivity of 58, conductivity of 1.15 S/m), sealed with 0.5 mm thick PDMS sheet (permittivity of 4, conductivity of 10 µS/m) and exposed to a background medium with variable conductivities (σ_B) to represent electrical loss from dry (1 µS/m) to under salty water (4 S/m) settings. The transmitter's power here is set to 560 mW to represent the maximum RF available power in the NFC reader chip here. These SAR simulations ignore NFC amplitude modulation and demonstrate the worst-case scenario because the peak emitted magnetic field of an NFC compliant device protocol may not exceed 10.5 A/m. (b) The transmission profile under various background losses demonstrates stable electrical characteristics that is significantly untied from environmental change. The SAR simulation, however, demonstrates decreased absorption rates with greater background conductivity as a larger fraction of the emitted electromagnetic power is dissipated in the lossy background medium. (c-e) Simulated SAR (shown

on the skin surface), surface electric current (on the metamaterial array), and magnetic field intensity (on the skin surface) at 13.5 MHz.

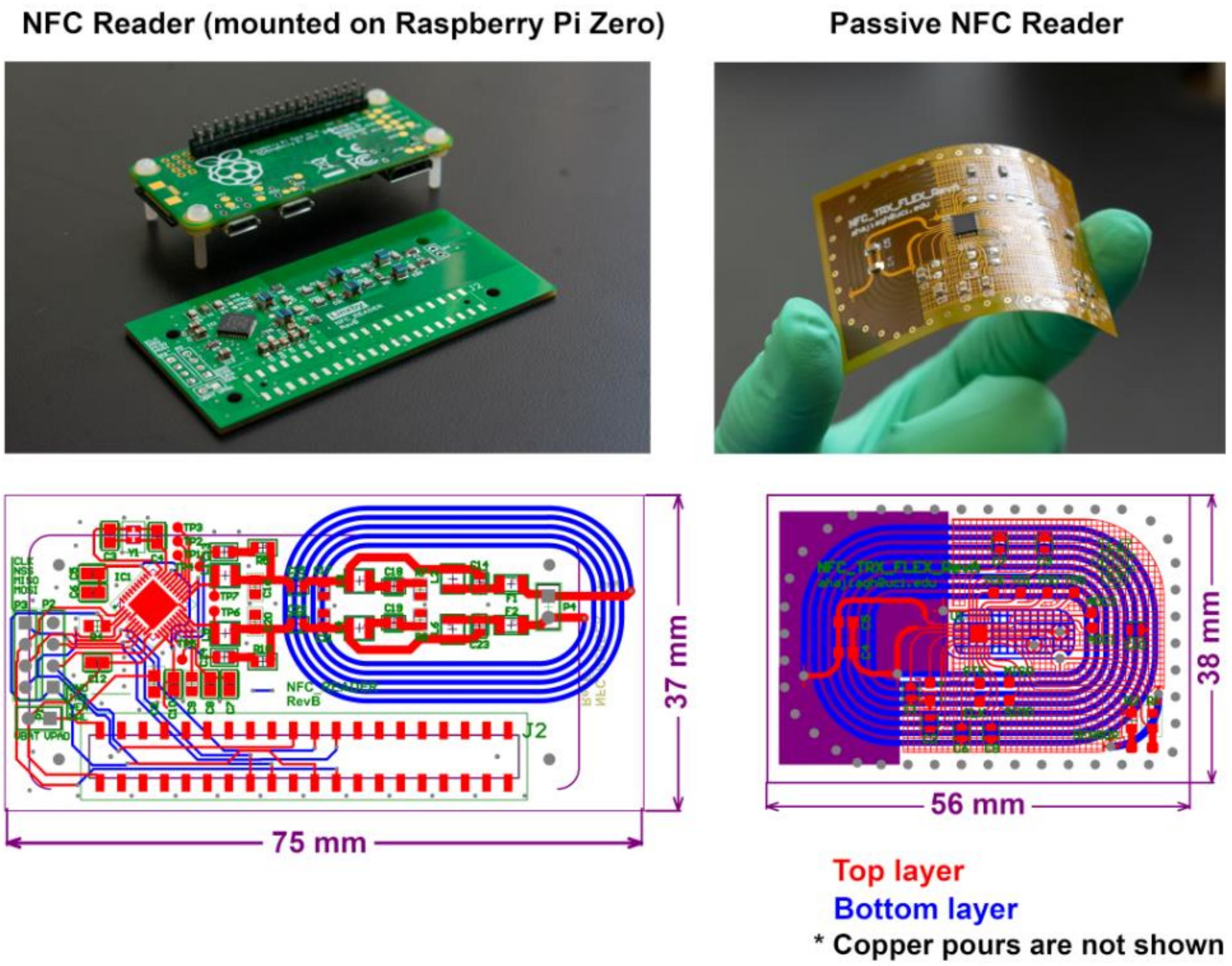

Supplementary Fig. 8. Active and passive custom electronic board designs.

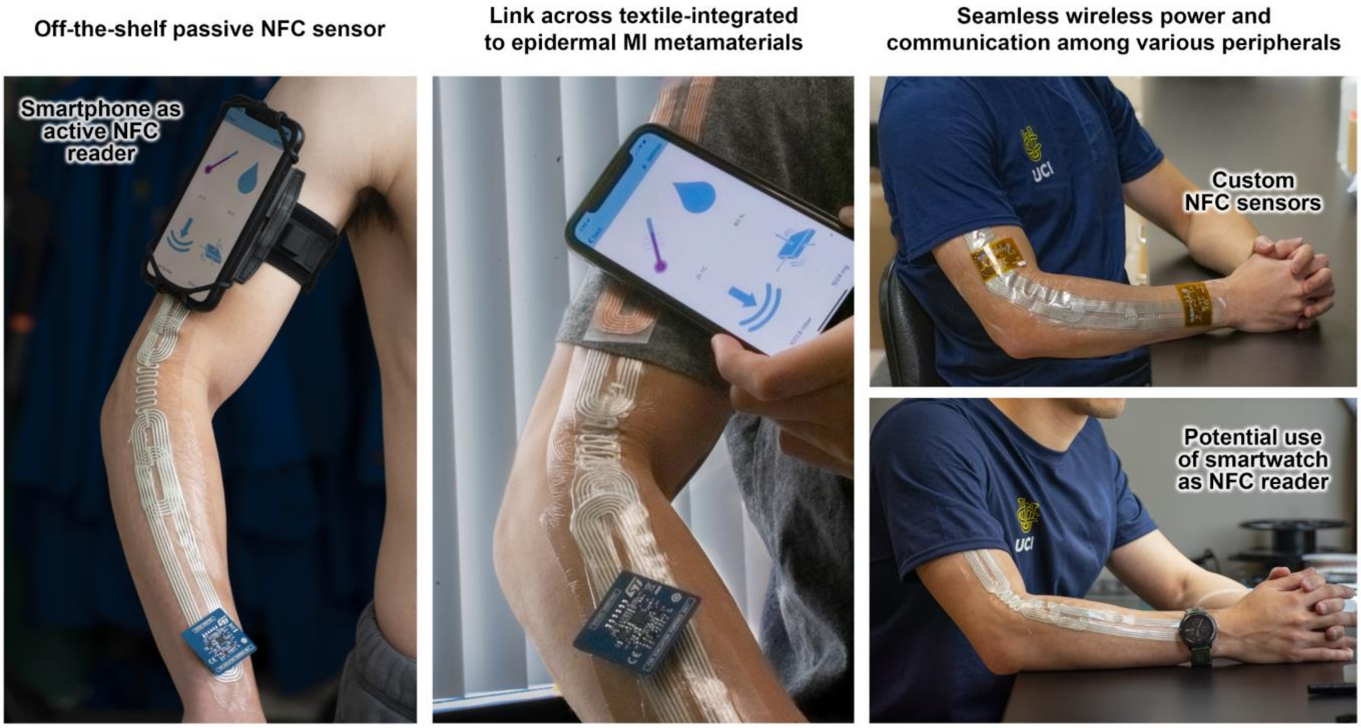

Supplementary Fig. 9. The nearfield propagation can be seamlessly routed along and between the epidermal and textile-integrated from factors, enabling multi-purpose applications.

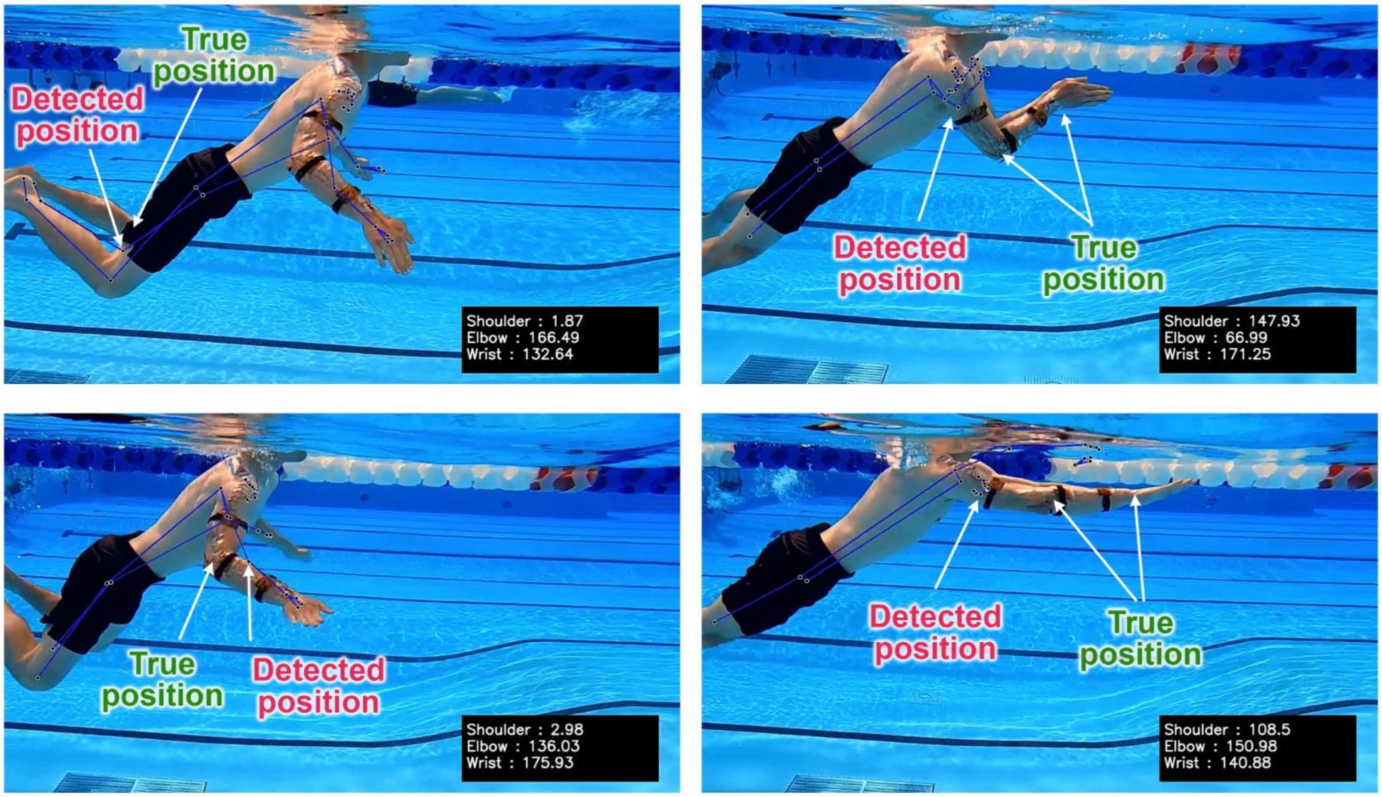

Supplementary Fig. 10. Misaligned camera position (compared to the dynamically moving body), in addition to environmental artifacts such as underwater reflections that interfere with the camera sensor often result in incorrect body angle detection.

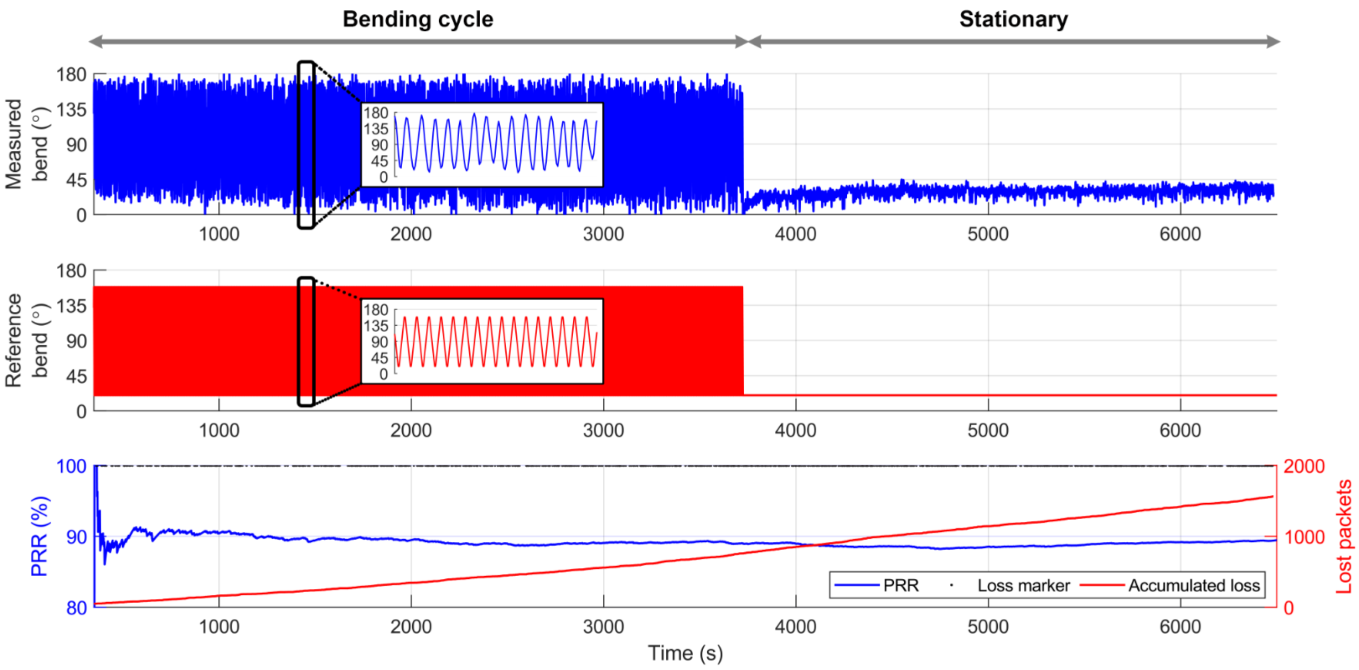

Supplementary Fig. 11. Long-term reliability of encapsulated underwater link. Long-term underwater network performance shows a stabilized 10.6% packet loss rate within 110 min of activity recording. For the long-time network characterization, we implemented a controlled continuous bending cycle on the strain gauge (connected to NFC sensor board) that is linked to the NFC reader through an underwater array of MI metamaterials. We stopped the bending after about an hour to ensure that the packet loss is caused by the network and not by the sensor board or strain gauge.

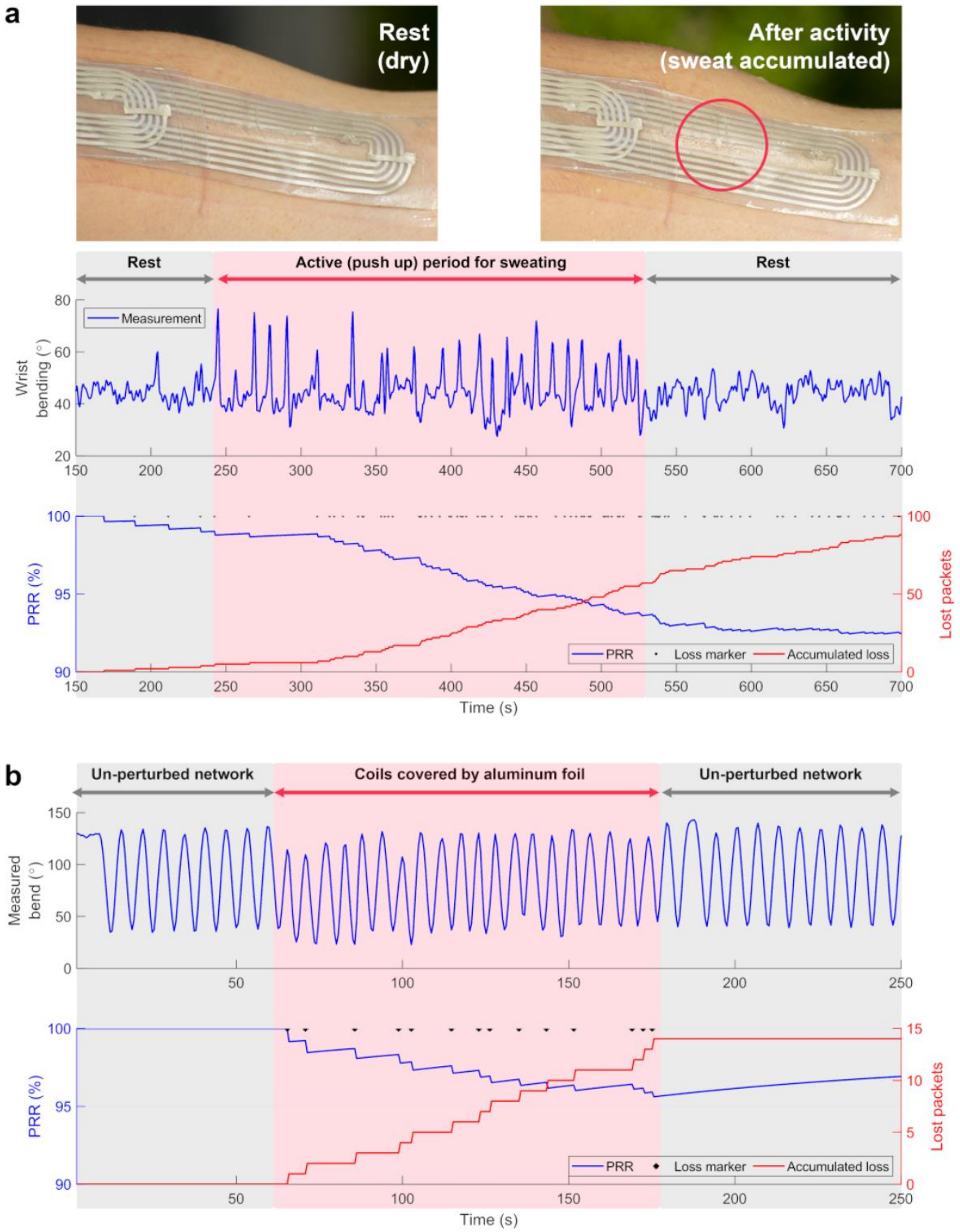

Supplementary Fig. 12. Environmental impacts on PRR. (a) We induced sweating through 5 min of outdoor pushups under 86°F, which is shown to impact the packet loss. The PRR slightly drops from 97% to 93% (from start to end of intense activity) which is primarily due to sweat droplets accumulated under the coils. This is comparable to underwater setting in which the network is surrounded in lossy media. Either way, the impact is expected to last until the network is completely dried. **(b)** To study the impact of surrounding objects (such as conductive threads or metallic peripherals) on the network performance, we recorded the PRR over 4 minutes while placing a standard piece of aluminum sheet covering about 30 cm2 of the dry encapsulated skin patches (highlighted from 60 to 180 s) on dry skin patches. This suggests a loss

rate of approximately 7 packets/min at a sampling rate of 2 Hz. Importantly, the sensor data demonstrates minor interferences, which may have been caused by the nature of amplitude shift key modulation employed in NFC protocol.

Method	Pro	Con	Architecture
**Wired (parallel)**	• Direct sensor integration	• Excessive connectors<br>• Multiplexing required<br>• Long wires<br>• Sensor chaining not possible<br>• Inadequate sensor sealing<br>• Challenging network modification	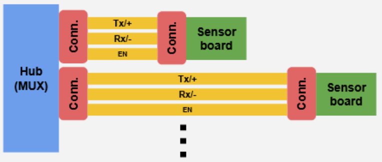
**Wired (serial)**	• Shorter/fewer wires<br>• Direct sensor integration<br>• Sensor chaining possible	• Excessive connectors<br>• Different sensor addresses required<br>• Inadequate sensor sealing<br>• Challenging network modification	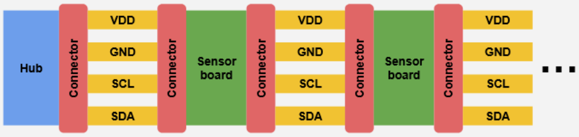
**Proposed wireless (MI channel)**	• No connectors<br>• No wires<br>• Fully encapsulated<br>• Sensor chaining possible<br>• Effortless network adjustment/extension	• NFC transponder required<br>• MI channel required	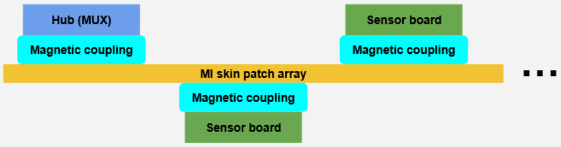

Supplementary Fig. 13. Comparison of hardware system integration with traditional wired and proposed wireless mechanism based on MI metamaterials.

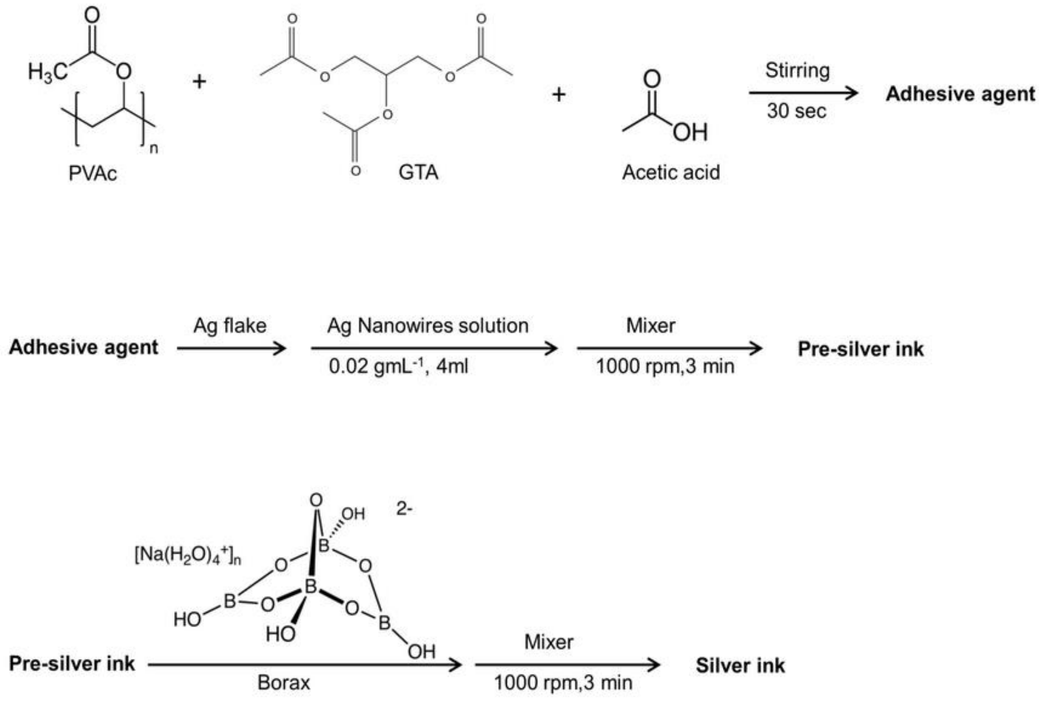

Supplementary Fig. 14. The waterborne-based silver ink synthesis process.

Supplementary Table 1. Landscape of available BANs.

Method	Wired[12]	Body-coupled capacitive[30,32]	Magnetically-dominant coupling[21]	Farfield radiation[16,33]	Clothing-integrated Nearfield propagation[37]	Skin-integrated Nearfield propagation (this work)
**Frequency range**	100 kHz - 1 MHz	< 150 MHz	100 – 200 MHz	2.5 - 5 GHz	13.56 MHz	13.56 MHz
**Pre-existing protocol compliance**	Yes (various serial buses)	No	No	Yes (BLE, Wi-Fi, Cellular)	Yes (NFC)	Yes (NFC)
**Power transfer capability**	Yes (µW to W range)	Yes (µW range)	Yes (mW range)	No	Yes (mW range)	Yes (mW range)
**On-demand network extension**	Difficult	Easy	Easy	Easy	Easy	Easy
**Path loss in proximity of body (dry setting)**	Ultra small (< 0.3 dB/m at 10 MHz)	Large (> 60 dB/m at 10 MHz)	Small (< 30 dB/m at 100-200 MHz)	Small (about 15 dB/m at 10 MHz; about 50 dB/m at 2.4 GHz)	Very small (< 18 dB/m at 13.5 MHz)	Small (< 25 dB/m at 13.5 MHz)
**Path loss in extreme (underwater) environments**	Ultra small (< 0.3 dB/m at 10 MHz with sealed connectors)	Non-functional	Large (anticipated > 30 dB/m at 100-200 MHz)	Non-functional	Non-functional	Small (< 25 dB/m at 13.5 MHz)
**Propagation security**	Bound to wires	Bound to body surface	Prone to third party listening in proximity	Prone to third party listening in far	Bound to body surface	Bound to body surface
**Versatility**	Compatible with standard	Specialized reader/sensor communication	Specialized reader/sensor communication	Compatible with standard	Compatible with standard	Compatible with standard
**Reader or sensor placement**	Only at the hub/terminals due to connectors	Anywhere	Anywhere	Anywhere	Anywhere	Anywhere
**Conformal to bodily stretches**	No	Yes	Yes	Yes	No	Yes (up to 120% strain when fully sealed)

Supplementary Table 2. Landscape of available conductive inks. The solvents utilized in the formulation of silver inks primarily serve as carriers for the ink's active components. A variety of solvents have been used for the fabrication of silver inks, including conventional organic solvents. In general, the organic solvent based silver ink has better stretchability and conductivity. However, in light of increasing environmental concerns and health-related considerations, water-based silver inks have been proposed as potential alternatives to environmentally unfriendly organic solvents and toxic chemicals. Here, we fabricate a silver ink which is not only water based but also with promised stretchability as the solvent based one.

Method	Waterborne silver flake (this work)	Solvent based silver ink[40–42]	Solvent based copper flake[57]	Carbon nanotube ink[58]	Graphene ink[59,60]	Conducting polymers[43]
**Bio-compatibility**	Yes	No	Based on solvent	No	No	Yes
**Conductivity**	Excellent (10^6 S/m)	Excellent (10^6 S/m)	Marginal (10^4 S/m)	Poor (10^3 S/m)	Marginal (10^4 S/m)	Poor (10^2 S/m)
**Stretchability**	Excellent (> 130% when fully encapsulated)	Excellent (140%)	Poor	Marginal (70%)	Marginal (74%)	Poor (30%)
**Solvents**	Water based (Elmer's glue/PVAc/Silver flake)	Organic solvent based (Silver-IPA complex/formic acid reductant/ethyl alcohol/PVP)	Solvent based	Organic solvent based (HPMC/SDBS/APTES/IUPAC)	Organic solvent based (IBA/Cyr/EC/AChE/ethanol)	Organic solvent based (P3HT/PVDF/APTES/PEDOT/PSS)
**Synthesis technique**	Easy	Difficult	Difficult	Difficult	Difficult	Difficult
**Cost efficiency**	Good	Good	Good	Poor	Poor	Poor
