# Supplementary material for: Amphibious epidermal area networks for uninterrupted wireless data and power transfer
Source: Nat Commun. 2023 Nov 18;14:7522. doi: 10.1038/s41467-023-43344-6 (PMC10657464; doi:10.1038/s41467-023-43344-6)
Supplement: Supplementary file 3 — Reporting Summary [file 41467_2023_43344_MOESM3_ESM.pdf]

nature portfolio

Corresponding author(s): Peter Tseng

Last updated by author(s): Oct 24, 2023

Reporting Summary

Nature Portfolio wishes to improve the reproducibility of the work that we publish. This form provides structure for consistency and transparency in reporting. For further information on Nature Portfolio policies, see our Editorial Policies and the Editorial Policy Checklist.

Statistics

For all statistical analyses, confirm that the following items are present in the figure legend, table legend, main text, or Methods section.

n/a	Confirmed	
☐	☒	The exact sample size (*n*) for each experimental group/condition, given as a discrete number and unit of measurement
☐	☒	A statement on whether measurements were taken from distinct samples or whether the same sample was measured repeatedly
☒	☐	The statistical test(s) used AND whether they are one- or two-sided<br>*Only common tests should be described solely by name; describe more complex techniques in the Methods section.*
☒	☐	A description of all covariates tested
☒	☐	A description of any assumptions or corrections, such as tests of normality and adjustment for multiple comparisons
☐	☒	A full description of the statistical parameters including central tendency (e.g. means) or other basic estimates (e.g. regression coefficient) AND variation (e.g. standard deviation) or associated estimates of uncertainty (e.g. confidence intervals)
☒	☐	For null hypothesis testing, the test statistic (e.g. *F*, *t*, *r*) with confidence intervals, effect sizes, degrees of freedom and *P* value noted<br>*Give P values as exact values whenever suitable.*
☒	☐	For Bayesian analysis, information on the choice of priors and Markov chain Monte Carlo settings
☒	☐	For hierarchical and complex designs, identification of the appropriate level for tests and full reporting of outcomes
☒	☐	Estimates of effect sizes (e.g. Cohen's *d*, Pearson's *r*), indicating how they were calculated

Our web collection on statistics for biologists contains articles on many of the points above.

Software and code

Policy information about availability of computer code

Data collection	The code supporting the NFC readout within this paper is based on NXP application note (AN12991) publicly available from NXP.<br>Electromagnetic simulations used the finite-difference time-domain method using commercial software (Comsol Multiphysics).
Data analysis	Mathematical analysis was implemented using commercial software (MATLAB).<br>The plots in this manuscript were generated using MATLAB. No custom data analysis was performed.

For manuscripts utilizing custom algorithms or software that are central to the research but not yet described in published literature, software must be made available to editors and reviewers. We strongly encourage code deposition in a community repository (e.g. GitHub). See the Nature Portfolio guidelines for submitting code & software for further information.

Data

Policy information about availability of data

All manuscripts must include a data availability statement. This statement should provide the following information, where applicable:

- Accession codes, unique identifiers, or web links for publicly available datasets
- A description of any restrictions on data availability
- For clinical datasets or third party data, please ensure that the statement adheres to our policy

All data supporting the findings of this study are available within the article and provided in the Source Data file. Any additional requests for information can be directed to the corresponding author.

Research involving human participants, their data, or biological material

Policy information about studies with human participants or human data. See also policy information about sex, gender (identity/presentation), and sexual orientation and race, ethnicity and racism.

Reporting on sex and gender	A single participant, a 30-year-old male, was included for the device demonstration in this study. The primary focus of this study does not pertain to statistical research in the medical or healthcare domain. Furthermore, characteristics such as age, gender, or similar covariates related to the participant do not influence the electromagnetic properties of the human body system, and as a result, they do not impact the study outcomes. Therefore, the inclusion of one subject is considered adequate for our purposes. The motion data gathered from the participant has been de-identified, and individual consent has been obtained for the sharing of this data at the individual level.
Reporting on race, ethnicity, or other socially relevant groupings	The human subject of this study is of Asian ethnicity. Race, age or ethnicity has not been used as proxies in this study.
Population characteristics	A single participant has been recruited for technology demonstration purposes.
Recruitment	The subject was recruited among UCI campus students. Their age and demographics may not impact the results of the study.
Ethics oversight	This publication was conducted in accordance with UCI IRB protocol HS# 2018-4843.

Note that full information on the approval of the study protocol must also be provided in the manuscript.

Field-specific reporting

Please select the one below that is the best fit for your research. If you are not sure, read the appropriate sections before making your selection.

☒ Life sciences ☐ Behavioural & social sciences ☐ Ecological, evolutionary & environmental sciences

For a reference copy of the document with all sections, see nature.com/documents/nr-reporting-summary-flat.pdf

Life sciences study design

All studies must disclose on these points even when the disclosure is negative.

Sample size	Sample size was determined based on similar studies in this field.
Data exclusions	No data were excluded from the analyses.
Replication	All experimental findings were reproduced reliably.
Randomization	Randomization is not applicable to this study since this research is not relevant to statistical study.
Blinding	Blinding was not relevant to this study since private information is not collected from the experiments.

Reporting for specific materials, systems and methods

We require information from authors about some types of materials, experimental systems and methods used in many studies. Here, indicate whether each material, system or method listed is relevant to your study. If you are not sure if a list item applies to your research, read the appropriate section before selecting a response.

Materials & experimental systems

n/a	Involved in the study	
☒	☐	Antibodies
☒	☐	Eukaryotic cell lines
☒	☐	Palaeontology and archaeology
☒	☐	Animals and other organisms
☒	☐	Clinical data
☒	☐	Dual use research of concern
☒	☐	Plants

Methods

n/a	Involved in the study	
☒	☐	ChIP-seq
☒	☐	Flow cytometry
☒	☐	MRI-based neuroimaging
